# “Cholera in New York” Criticised; the Quarantine Question

**Published:** 1888-02

**Authors:** Arthur S. Wolff

**Affiliations:** Quarantine Officer at Brazos Santiago; Brownsville


### CORRESPONDENCE.

### "CHOLERA IN NEW YORK" CRITICISED; THE QUARANTINE QUESTION.

LETTER FROM DR. A. S. WOLFF, QUARANTINE OFFICER AT BRAZOS SANTIAGO.

*Editor Daniel's Texas Medical Journal.*

DEAR SIR:—It is with reluctance that I intrude on your valuable space, and time of your readers. But I think I have something to say with reference to your "leader" of the October number, on the subject of quarantine. Of course I am well aware of the disadvantages of a contest in which you will have the dying speech. Still I am determined to cross swords with you. I won't mind a few slashes when dealt by Dr. Daniel. Of course it will be the story of the "eels under the process of skinning." But it is nothing when you are used to it."

It is scarcely necessary to enter into the historical details upon the subject of cholera, or upon the controversy respecting the manner in which the disease is propagated. The subject of cholera in all its relations has, from its approach to our shores ,become of pressing importance, and notwithstanding all our best efforts, we may not succeed in keeping the enemy at bay; he may break into our camp, challenge us to a hand to hand encounter, which, should your advocacy of the English system be here followed, lead us to disaster and to which I enter my protest.

To be forewarned is to be forearmed. The disease is not one which overpowers us at once. It has to be introduced from infected places, more or less directly. Its arrival has been foreseen and foretold, moving onward, under extreme temperatures and climates, in the teeth of adverse winds, through heat, cold, moist or dry atmospheres. I confess it is difficult to understand why a gentleman holding a dignified rank in the medical profession, and the editor of one of the most popular medical journals in the country, should adopt the unscientific nonsense of a Dr. Thorne; it is a puzzle to me.

The subject of quarantine has some anomalous features. No analysis made up to date by our best sanitarians, however comprehensive, can furnish evidence which would satisfy all. Unless some one takes it in his head to turn a Prometheus, and make new men, he can hardly devise a quarantine scheme which would be satisfactory to the interest of all countries who have intercommunication and commercial relations. But the question is of deep importance. It not only involves the general safety of mankind, but it is intimately connected with the commercial welfare of nations. For, as it has been truly observed, if cholera is not carried and propagated, quarantine laws are absurd, and commerce needlessly burthened. The establishment of lines of circumvallation, guarded to confine from the yet uninjured, and the fears it necessarily induces, are elements as dangerous in character to the community as they are pernicious in their effects to the common feelings of humanity. But on the other hand, if the doctrine be true to the extent of our most accurate observers, that the disease is carried by human beings, ships, cargoes, clothing, etc., then the quarantine restraint cannot be too rigidly enforced, nor can the conduct of speculative theorists be too severely reprehended lulling the ignorant and unwary in false notions of security.

Having introduced my subject, I hope, in a dignified manner, I proceed:

"THE CHOLERA IN NEW YORK."

I premise by asking a fair space, and some latitude, for the reason that I shall be obliged to be somewhat prodigal in quoting the English law, in the criticism of your vaunted English quarantine system.

You say: "When we reflect that it is a truth beyond dispute, that the quarantine *alone* has never barred the March of cholera." This is a bold assertion, but the word "alone," of course, will give you a fair opportunity to get you out of that non-committal expression; and here I take issue with you by stating that it is established beyond peradventure, that cholera has never gained a foothold anywhere, but where the quarantine was a paper one.

"France, Italy, Algeria, Spain, all quarantine countries. Yet see what sad havoc the plague had made: Spain alone lost 119,620 of her habitants."

The above named countries have no doubt tried to ward off cholera by quarantine restrictions. But of what avail are good intentions when the resources are inadequate and insufficient, or the agents charged with carrying out the preventive measures are notoriously corrupt.

Now let us inquire how the cholera was introduced in Europe in 1884.

I suppose you will admit that the Sarthe arrived at Toulon on June 4, 1884, with clean bills of health, although four of her crew died of cholera before she sailed, and that cholera prevailed a Saigon, the point of her departure.

"We know that there was a small epidemic of cholera at Marseilles in 1883, and that it was carefully concealed, the doctors and nurses having been sworn to secresy by the authorities. Where this cholera came from is not positively known, whether from Tonquin, China or Egypt, as the particulars are still purposely shrouded in darkness." The preceding, and what follows, I quote from Edmund Ch. Wentt, M. D., on Cholera, 1885, page 67."

"The first we heard of cholera in France for a long time was at Toulon early in June. Transports from Tonquin with the disease arrived, and its presence was concealed for at least three weeks, until it could be hidden no longer. Then the early records were not only lost, but shamefully concealed. The consequence was the tremendous panic with which all are familiar. At least 10,000 or 15,000 Italian laborers fled from Toulon, some to Marseilles, and many more to their homes in Italy. The epidemic broke out at Marseiles so soon after it was announced at Toulon that some doubts have been raised whether there was recrudescence from the fall epidemic, or whether it was brought from Toulon. The earliest cases seemed to be refugees from Toulon, but no one knows whether still earlier cases were concealed until the blame could all be put on Toulon."

"Cholera had been prevalent in the French army and fleet at Tonquin, that their operations were seriously interfered with, and of course the French authorities concealed all they could about it for political and military reasons. But the infected transports did come from Toulon. The same panic arose at Marseilles, and at least 10,000 or 15,000 more Italians fled to Italy."

"The quarantinc on the northern border of Spain was effective, till a family from Marseilles, wishing to escape quarantine, went to

Algeria where there was no cholera yet, and sailed for Alicante on the coast of Spain, and started the epidemic."

"The Italian refugees from France slipped through the mountain passes and brought the pestilence to the western provinces, (viz.) Turin and Cuneo. The Italian government sent transports to carry off others, which were landed principally at Genoa, Massa, Carrara, further down the west coast, and especially Naples. Rome refused to receive any refugees, and escaped the pestilence. Italy became impregnated, and hence the epidemic."

Now, friend Daniel, is that the kind of quarantine Dr. Thorne refers to, when he and you assert that in quarantine "countries it has always failed?" Why, the assertion is too futile to answer, and you know it; at least, I hope so.

See what Stille, page 755, affirms: "There is urged against the enforcement of rigid quarantine by land and seas the singular arguments that it has not always excluded the disease. A more logical inference would seem to be, that since it succeeded, not completely, but partially, its inefficiency should be charged to its imperfect execution. Or even granting that absolute exclusion of cholera is impracticable in every instance, does it follow that the transit of men and things should be unimpeded? This is taking counsel from despair—is a stupid fatalism, which one might imagine to have been imported with the disease from the East!" It is difficult to characterize that state of mind which concludes against the use of a salutary measure. because its efficiency is not absolute; the more so, when it is true, and admitted that its inefficiency is not intrinsic, but due to negligent and fraudulent administration.

I stand on the record, and assert, where there has been failure, it was, and is due to laxity of the observance of rigid quarantine measures.

"Tommasi Crudeli, of Milan, writing after cessation of the cholera at Milan 'Sea quarantine is easy to violate, too many interests prompt its violation, and it is seldom that people, as did the Sicilians, unite in silencing such interest.'"

"In Sicily, in 1865, the whole population advocated quarantine, and absolute non-intercourse with infected countries, and the island was thus guarded until the insurrection, September, 1866. The epidemic, the very day on which the troops were sent from Naples, where cholera was raging, arrived at Palermo, spread rapidly, and the incident shows that when sustained and rigidly enforced, sea quarantine is efficacious."

The Honorable Philip Carroll, U. S. Cousul at Palermo, writing to Honorable William Hunter, Second Ass't. Secretary of State, under date of February 9, 1885, says: 'With reference to the non-appearance of cholera in Palermo during the recent epidemic, in places almost conterminous thereto, the general belief here is, that the immunity was due to the effective quarantine which was at once established and rigorously maintained until some time after it was suspended in other places, and every vestige of the disease had disappeared. In support of this belief they quote the epidemics of 1865, 1873 and 1884. When vigorous and rigid measures were enforced and maintained they escaped the plague. Whereas, in 1866 the insurrection broke the quarantine and made sad havoc among the habitants."

Let me call your attention how quarantine is observed at some stations.

Omar Pacha in 1884, bearing a message to Constantinople concerning the Suez Canal. Three persons died of cholera on the passage. The physicians and the captain concealed the deaths at the Pacha's order, and declared the ship healthy to avoid the quarantine. Thousands fell victims to the disease.

The quarantine was inefficient, and too late. Dr. Mosa, *Le Precantione contre il Colera e le quarantine*, states; "when I asked Dr. Koch what he thought of the quarantine in operation in Italy, he replied, I am convinced they are half measures, worth nothing, and such quarantine are without result."

Of course they are, I claim that the whole measures are. The opinion of Dr. Thorne, notwithstanding.

You state on the information of Dr. Thorne, no doubt.

"From Toulon in June cholera spread to Marseilles and other French cities; thence to Italy, Algeria and Spain. These countries imposed quarantine measures." May I ask by what process of reasoning you came to the conclusion that the named countries did maintain a rigid quarantine? Is it because Dr. Thorne said so? I challenge him or any one else to produce the slightest evidence that quarantine was enforced, in time to prevent the plague entering those countries, and it has been proved beyond cavil to the contrary, the opinion of your English sanitarian.

Now let us consider the English quaratine on which you pass such high panegyrics, and for which our health officer should abandon a system recognized by all civilized nations as their only safeguard and protection.

In Great Britain, Ireland and Wales, the quarantine law passed in the reign of George IV., Capt. 78, and provided for a rigid quarantine. The law has been repealed.

In 1866, the sanitary act was passed. Under this act, every city, borough, district, parish, township or hamlet, appoints inspectors of public health. To this act was added the Statute 14th and 15th, Victoria, Capt. 68, appointing medical officers. The whole kingdom has a net-work of auxilliary sanitary boards.

With some modifications came the acts of 1879 and 1882, the outcome of the Vienna Conference of 1881, under which your Dr. Thorne's quarantine is carried on in England, with the knowledge that the members of that very conference have reversed their opinion, and claim that rigid quarantine measures is the only safety for the people.

But even the 1887 Conference does not justify the quarantine measures he employs to-day.

Transactions International Medical Congress, Vol. IV, p. 419, London 1887.

"It is natural that diversity of climate and different seasons, should modify the practice of quarantine at particular ports, but there should be no laxity of administration at anytime or anywhere. The medical inspection which is the only method of ascertaining the facts regarding the sanitary condition of passengers, cargo, crew and ship, should be everywhere carried on in the same way, carelessness in making such inspection is a crime and should be so considered; efficiency depends upon honesty, faithfulness and patriotism of the inspector. No man should be elected for this great responsibility who does not combine with the necessary technical knowledge, these three characteristics."

No doubt but Dr. Thorne considers all the English sea ports, "particular ports." The rule adopted by him is very suggestive and significant, under which he acts, and is as follows:

"All vessels on which infectious disease manifest itself during the voyage to the United Kingdom, shall immediately, if South of Cape St. Vincent, repair to *some* Lazaretto in the Mediteranean, *there* to perform quarantine, and if to the Northward of Cape St. Vincent, she shall immediately repair to Milford Haven."

You will admit that is an easy way of disposing of their infected ships, let other countries take care of them, and it is not a bad scheme. The local Government Board got another act passed July 12, 1883, modifiying all previous acts and quarantine rules. This act originated through the representations of the agents of commercial corporations. This board is constituted of merchants, ship owners, and others interested in the welfare of English maritine commerce. I don't suppose that there was much difficulty in finding an anti-quarantine doctor willing and ready to carry out rules formulated by the very men to whom he owes his appointment.

See what Dr. Quain says about this matter: "Quarantine as carried out now, has no longer a medical signification. It is practiced to a limited extent, solely with the view of relieving our maritine commerce."

Dr. Mapother, page 617, etc., etc.: "Any ship or vessel coming to this country, or lying in river or harbor—shall be subject to the jurisdiction of the nuisance authorities. But this section shall not apply to her Majesty's ships." Of course a ship of war is innocuous, and is proof against infectious diseases. Where Dr. Thorne got that inspiration from, is too insignificant to inquire into, but it is certainly errant trash.

However, 1872, the danger from the importation of infectious diseases, was so glaring and apparent, that the English Government, could not without danger ignore public clamor, and appointed Dr. Mapother to make inquiry about the system of quarantine then in force; he reports to the Privy Council as follows: "Compelling the removal to quarantine hospitals all ill from cholera, absolute detention, fumigation and disinfection, of everything, passengers, clothes, cargo; or nothing short of stopprng all maritine intercourse with infected countries can be relied upon for keeping out cholera."

Did England act upon this very intelligent report made by one of their most advanced sanitarians. The author of one of the best works on Hygiene? Certainly not.

The merchant princes representing large East Indian interests, fearing that their enormous gains were going to be interferred with, readily found an anti-quarantine advocate to help them out of their dilemma, and Drs. Mapother and Farr's report, remained a dead letter. A quarantine interference would be too costly, and means must be found to break the force of this report, or somehow to evade rigid quarantine measures as advocated by this great report:

The first move was the report of Dr. Cunningham, the Sanitary Commissioner with the Government of India, forbidding the head of departments, to state or even hint in their reports, that cholera is carried from place to place, and he persists in following the steps of his predecessor, Sir J. Strauchey, affirming that cholera is not, or ever will be a communicable disease."

This was heeled by other reports, among them the following: "The British Consuls, at Kobe and Osake, Japan, wrote to their Minister, to the effect that the disease which raged in Japan, was not Asiatic Cholera, at the same time deprecating quarantine measures, and that in the face of an appalling death rate, the disease then was of an unusual malignant type, thousands fell victims to it. England knows, ship owners and merchants dread the obstrution of trade, were the truth to go abroad."

No my friend, the English system of quarantine is no quarantine at all, and could only originate and advocated by commercial corporations or joint stock companies who care nothing for the health of the people, provided, they get their cargoes on the market when on the "boom."

I am one of those who believe and think it of more importance that the meanest wretch in the State of Texas should live in safety, even if all the cargoes of the commercial lords should wait a twelve-month or to "dooms-day" if a rigid quarantine will save him.

England has based her loose quarantine on two doctrines, both absurd in the face of the facts as we know them to exist.

"England is in commercial intercourse with all nations, and in constant communication with the different countries of India in which cholera is always found, and yet, she has never brought home directly a case of cholera."* Will you dare to father such a palpable uatruth? But supposing it is true, will that make any difference to us here?

If she has never brought home a case of cholera, remember that ships south of Cape St. Vincent must find an asylum in some *Lazaretto* in the Mediteranean, and other nations are burdened with the infected ships. But further, supposing she had not imported a single cholera case from India, will that invalidate the fact that the path of many of her ships from Calcutta or Bombay is strewn with the victims who died of cholera, before they arrived at "Port Said," and, of course, from there to England is at least fourteen days, and if these are passed without appearance of symptoms of cholera on board, there is no danger.

The other doctrine of which your Dr. Thorne is a strong advocate and supporter, is: *That the places in which cholera is endemic* are dangerous only when the disease happens to exist in an epidemic form. A convenient doctrine you will admit.

Let me here quote *The Gazette Hebdomadaire des Sciences Medicales*, July 26, upon this subject. In his address before the Academie of Medecine, Dr. Fauvel said: "Of late years the commercial spirit of English authorities in India has prompted them to devise a doctrine very convenient to themselves, and one, that enables them to avoid the inconvenience of quarantine at Suez. This doctrine considers places where cholera is endemic, as dangerous only when the disease happens to exist in an epidemic form." Is it necessary to impugn or contradict such an absurd position? I think not.

But she was caught in her own trap on several occasions. Let me give you one or two instances as illustrations:

"The steamer 'Mira,' from Calcutta, on April 21, 1886, lost one of her crew before touching Colombo, and she reached 'Suez'—sixteen days had elapsed—all on board were well. Still she was ordered to 'El-Tor,' performed seven days quarantine, was fumigated and disinfected." It would not do in 1886 to let her go on, until she reached Cape St. Vincent, and then go to some *lazaretto* in the Mediteranean, because, she would be refused refuge, and not daring to proceed to England with her history, she had to swallow her own medicine.

* Dr. Wolff misquotes Dr. Thorne. Ed.

"In 1883 cholera was declared at Damietta. A commission of physicians was sent from Alexandria on the 25th of June, and the disease was declared an epidemic of cholera. The British consul, who is a member of the Egyptian Sanitary Council, insisted that the disease was of local origin, and takes pride in saying: 'That he had fought many battles with his colleagues in the interest of British ship owners, and their vast English interests in the East.'

"The Enlish government still denied the disease at Damietta being cholera, although it was very fatal, sent their Surgeon General, one of the most determined anti-quarantine advocates, to examine if the disease was really cholera, and without affirming or denying it, makes the extra-ordinary statement: 'That cholera has never been imported from E. India to Egypt, and that cholera is a local malady.' Dr. Wendt, page 65, on cholera.

Dr. Guy, page 78, strikes the key-note of the whole of the English quarantine business. "To a great mercantile nation like ours quarantine leads straight to troublesome restrictions, to the great detriment of our shipping interests, and this consideration has certainly blunted the perceptive faculties of our anti-quarantine advocates." Is it necessary to go on and heap more evidence on the subject, to prove that the English quarantine, as carried out by your Dr. Thorne, is a farce, and that it is notoriously subjective to your cotton, coffee and tea lords, the whole of the machinery must always give way to her mercantile interest.

I hear you say, this criticism is getting monotonous. Well, do you wish the pages you devote to the subject to remain uncontradicted? You say: "The danger, the great wrong, and the great injustice and inhumanity consists in keeping those hundreds of well persons together. New York should rise up, etc., ets., etc." Now, friend Daniel, I can hardly reconcile to myself that such nonsense came from your pen, unless your credulity has been played upon. You ought to know that such a thing never can, nor ever did happen anywhere at American quarantine stations in modern times, and you ought to know better. But it evidently does happen in England, or Dr. Thorne would not make a point of it. I willingly join you in expressing the abhorence and odium that should be attached to such a practice.

You go on: "We do not see how the sanitarians of this government, in knowledge of the facts above set forth, can hesitate to act at once, set in operation the plan, the only plan, which has repeatedly been demonstrated to be efficient. It is absurd to say that the medical officer in charge, Dr. Smith, who is an advanced thinker, and a recognized sanitarian of high order and ability, etc., etc." "Would he blindly or willfully shut his eyes to the lesson taught by failure of quarantine in other countries, and the success of the English plan, etc., etc."

To this puff indirect to Dr. Smith, I answer: That all the best sanitarians in the State of New York entered their protest against the quarantine management as carried out by Dr. Smith at the New York quarantine establishment.

See the letter of Mayor Hewitt, of New York, to the Governor of the State, in which he calls attention to the disgraceful state of affairs at the quarantine station, and refers to the fact, that the sad disclosures recently made are such, as to endanger not only the State of New York, but every State in the Union, and asks the Governor his immediate action, and inquire into Dr. Smith's quarantine management.

The Academy of Medicine, appointed a Committe of Inquiry in the quarantine management of the State of New York. The committee was constituted of such men as Drs. Agnew, Janeway, Stephen Smith, A. Jacobi and Richard H. Derby, who, after a careful inquiry in the quarantine management as carried on by Dr. Smith, qualify it as disgraceful, as it is contrary to all principles of modern experience.

Other medical and sanitary organizations have joined the above in the protest and asking this day the legislature to save the people from the apparent danger to which they are threatened. This does not work very flattering for your advanced thinker, does it? Nor does it show that he is a "sanitarian of high order." In all fairness, does it now? The opinion of those gentlemen is such as to warrant the belief that they have no high opinion of him.

You are struck with holy horroy because someone suggested, that the authorities should have turned back to sea the "Alesia," "which would have been to condemn them to a certain fate. such an act would have been in violation to common laws of humanity, such an act would shame a savage. etc., etc., etc."

Why, this is the very thing your English quarantine authorities did, and of course, will always do, if it is for the best interest of their commerce.

Here are a few instances: "The story of the Helvetia, as told by Dr. French. The vessel sailed from Liverpool on May 2, with German emigrants, during the following day the cholera appeared among them, she went to Queenstown for refuge, the authorities refused to grant it, and the vessel returned to Liverpool. The danger of infection was thrown back were it belonged.

England, by proclamation in 1884, refused entrance in the harbor of Malta, and ordered all French vessels to quit the port, and further orders were issued, that ships from France or Italy be not admitted in the port of Malta. Ships so arriving were compelled to go to sea.

Portugal, in 1884, had the same rule. The ship St. Marc arrived off Lisbon; some distance out she was inadvertently boarded by some Portugese officers. The ship was not allowed to communicate, and refused pratique, with peremptory orders to leave; the officers had to remain on board, and took the journey back on the St. Marc.

MADEIRA IN 1884.

In July, several vessels coming from French or English ports were refused entrance. and returned to the ports from whence they came, Of course, in your opinion, if this was done in America it would "shame a savage." England, Portugal, and other countries have so acted, therefore the quarantine authorities of those countries have performed actions "to shame a savage." Yet I can see nothing so very inhuman in refusing an infected ship in our ports, under certain circumstances, certainly not if they went to infected ports for cargo.

"On 6th of December, 1849, a ship arrived from Hamburg with 250 passengers, after a passage of fifty-five days. Cholera was prevailing at Hamburg when the ship sailed. Six or seven cases occurred on board of the vessel while she was still laying in the river Elbe." See Tardien, page 218, on cholera.

In 1872, two ships, one for New York; and one for New Orleans, brought emigrants to both cities, and these emigrants were taken from infected ports with the full knowledge of owners, agents and captains. Let me ask you where is the wrong in refusing entrance to such ships? I opine that to leave a country with a lot of infected material, and for the sake of profit, get a cargo and human beings, start with them, make the ship a Lazaretto by the time her journey is at an end, and infect, with " malice " prepense, a whole country, is indeed a crime that should "shame a savage."

Why should not a ship with cholera on board not return from where she hailed? The captain, agent or owner knew they were loading an infected cargo, but when they find that this load of infection cannot be delivered or landed, they will take care to avoid infected ports, and no fear of importing infectious diseases need be entertained by sea.

However, it is one thing to impose quarantine restrictions, and another to enforce it. The danger is that some weak point will be neglected and that the scourge will find an entrance somewhere. The fault is to be found not so much in the undertaking than in the undertaker.

As a State we are in much less danger than many others, but eternal vigilance is the price of our safety. No, my friend, the English system of quarantine will never do here, for the reasons above mentioned. The safety of the people depends on a rigid quarantine, with an inspection, isolation, disinfection, etc. The people of the State of Texas demand it, and shall we abandon them when the disease may be at our doors to-morrow? Never! Not for all the ships and cargoes in the world. The ethics of all civilized nations have long adopted, as an action, the principle that in the conflict between public health and commerce, precedence must be given to the former.

The policy of paliation, in which we have so long indulged, must be abandoned. It has been found eminently unfruitful, and in many instances mischiveous.

The policy of prevention is the rule of action for the sanitary legislator.

" It is the business of the sanitary legislator and administrator to give due recognition to those natural laws, in their endeavor to maintain the health and save the lives of the people, and as the knowledge of the causes become precise, and the technical means of prevention more efficient, public health will be steadily improved with the advance of wise legislation."—Dr. Parker.

The public is awaking to the importance of the question. The occasion is highly favorable, the daily press, just at present, is lending a helping hand, and why the editor of the leading medical journal of Texas should object, or adversely criticise the system of quarantine as observed and carried on by the health officer of the State of Texas and urge a system of quarantine measures of not the least utility or protection to the people, is "one of those things no fellow can make out."

I have no suggestions to make; the health officer at the head of our quarantine affairs has long ago instructed his subordinates, that it is their duty to secure safety to the people, with the least interference with commerce or travel, but vigilence and rigid observation of quarantine measures must be observed and carried out where called for. I know that up to date this has been accomplished under many difficulties and draw-backs, but worse than all, with inadequate means to do what the important and great work demands.

Please remember that the State of Texas would hardly put at the disposal of her health officer $80,000,000 or $400,000,000 for quarantine purposes; all that can be done with the small appropriation at his command is done, much more remains to be done. But, alas! where is the money to come from.

The land quarantine will be considered anon; and in the mean time I hope that my reply to your leader will not leave the impression that I have some mercenary motive. I hope to reap no further reward than my conscience affords. ARTHUR S. WOLFF., M. D.

